# Botulism outbreaks in natural environments – an update

**DOI:** 10.3389/fmicb.2014.00287

**Published:** 2014-06-11

**Authors:** Mari Espelund, Dag Klaveness

**Affiliations:** ^1^Protection and Societal Security Division, Norwegian Defence Research EstablishmentKjeller, Norway; ^2^Department of Biological Sciences, University of OsloOslo, Norway

**Keywords:** *Clostridium botulinum*, botulism, serotype, spore, anaerobe, lakes, wetlands, soil

## Abstract

*Clostridium botulinum* comprises a diverse group of botulinum toxin-producing anaerobic rod-shaped spore-forming bacteria that are ubiquitously distributed in soils and aquatic sediments. Decomposition of plants, algae, and animals creates anaerobic environments that facilitate growth of *C. botulinum*, which may then enter into food webs leading to intoxication of animals. Via saprophytic utilization of nutrients, the bacteria rapidly sporulate, creating a reservoir of highly robust spores. In the present review, we focus on the occurrence of *C. botulinum* in non-clinical environments, and examine factors influencing growth and environmental factors associated with botulism outbreaks. We also outline cases involving specific environments and their biota. In wetlands, it has been found that some *C. botulinum* strains can associate with toxin-unaffected organisms–-including algae, plants, and invertebrates–-in which the bacteria appear to germinate and stay in the vegetative form for longer periods of time. We suggest the need for future investigations to resolve issues related to the environments in which *C. botulinum* spores may accumulate and germinate, and where the vegetative forms may multiply.

## INTRODUCTION

The species comprises multiple highly heterogeneous strains of rod-shaped anaerobic spore-forming bacteria, which are categorized into four groups (Groups I–IV) based on genomic relatedness. All *C. botulinum* strains produce botulinum toxin, which paralyzes animals by inhibiting acetylcholine release from synaptic vesicles at neuromuscular junctions. This toxin is classified into eight serotypes designated A–H (Collins and East, 1998; Barash and Arnon, 2014), of which A, B, E, and F are shown toxic to humans. Botulinum toxin-producing bacteria are divided into six groups: *C. botulinum* Groups I–IV as well as some strains of *C. baratii* and *C. butyricum* (Peck, 2009). Group I includes the proteolytic *C. botulinum* strains that produce botulinum toxin serotypes A, B, and F. Group II comprises non-proteolytic strains that produce toxin serotypes B, E, and F. The strains in Group III produce serotypes C and D, or mosaic C/D toxins. Group VI strains, referred to as *C. argentinense* (Suen et al., 1988), produce toxin serotype G. Among the other species, *C. butyricum* produces botulinum toxin serotype E and *C. baratii* produces serotype F (Hill et al., 2009).

Botulinum toxin genes exhibit remarkably variable organization. They can be chromosomally localized or localized on plasmids or phages (serotypes C and D). Serotype B transcription can occur through both genome-encoded and plasmid-encoded toxin gene clusters (Franciosa et al., 2009). Genome comparisons have revealed evidence of toxin cluster evolution through horizontal gene transfer, site-specific insertion, and recombination, and genomic analysis has supported the historic group classifications (Hill and Smith, 2013; Stringer et al., 2013). Thus, the factors affecting pathogenicity are apparently subjected to a higher evolutionary rate than the core genomes, allowing for fast environmental adaptation of the pathogen.

The ecology and properties are similar enough among Groups I–IV that it remains meaningful to discuss *C. botulinum* in the environment as a single group. *C. botulinum* spores persist in soils and aquatic sediments for decades, and propagate by predator-dependent disease transmission. Upon entering the food webs of animals, *C. botulinum* toxins may intoxicate and kill the animal, or infect and proliferate and kill the prey. Saprophytic utilization of the prey via enzymes, including proteases and chitinases, makes nutrients available for massive spore and toxin production. Neurotoxin gene expression and toxin complex formation reportedly occur in the late exponential growth phase and the early stationary phase (Bradshaw et al., 2004; Kouguchi et al., 2006; Artin et al., 2008; Cooksley et al., 2010), and toxin production and sporulation seem to be co-regulated (Cooksley et al., 2010).

It appears that contaminated soils and sediments are primary environments for spores and serve as an incubation area, from which the pathogens may be mobilized (Long and Tauscher, 2006). *C. botulinum* is detected in, or may be associated with, various organisms that are not affected by the toxins—such as algae, plants, and invertebrates (Quortrup and Holt, 1941; Duncan and Jensen, 1976; Bohnel, 2002). Fish are carriers of *C. botulinum*, but botulism outbreaks in fish populations may lead to death on a large scale (Yule et al., 2006; Hannett et al., 2011). Avian botulism caused by *C. botulinum* type C, mosaic C/D, or E is a common cause of death among waterfowl (Skulberg and Holt, 1987; Friend, 2002; Takeda et al., 2005; Lafrancois et al., 2011; Vidal et al., 2013). Unpredictable outbreaks with variable losses have been reported worldwide (Friend, 2002; Babinszky et al., 2008; Shin et al., 2010; Vidal et al., 2013). In recent years, large outbreaks in the Great Lakes, with high mortalities among fish and birds, have been well documented and analyzed (Perez-Fuentetaja et al., 2006, 2011; Lafrancois et al., 2011; Chun et al., 2013). In this review, we discuss factors related to botulism outbreaks in natural environments.

## ENVIRONMENTS AND REGIONS

*Clostridium botulinum* is ubiquitously present in the environment in soils, dust, and the marine and freshwater sediments of wetlands, rivers, and lakes. Spores in soil may be mobilized by surface waters in heavy rain, or dust carried away by wind (Long and Tauscher, 2006). Botulism has been characterized as a particularly substantial risk to humans in northern climatic regions, due to intoxication from poorly preserved food (Dolman, 1960; Hauschild and Gauvreau, 1985; Austin and Leclair, 2011; Fagan et al., 2011; Leclair et al., 2013b). Serotype E is dominant in sediments of the arctic and subarctic regions, whereas serotype B is most prevalent in soil (Johannsen, 1963; Miller, 1975; Huss, 1980; Hielm et al., 1998; Leclair et al., 2013a). The temperate climate zone of Europe shows the same distribution pattern, in which serotype B is most prevalent in soil and serotype E is found in sediments (Huss, 1980), although serotypes C and D are also commonly found (Woudstra et al., 2012). In the temperate zone of Northern America, serotype A is most common west of the Mississippi river, and serotype B east of the Mississippi river (Shapiro et al., 1998), whereas serotype E is most common in the areas of the Great Lakes and the Pacific Northwest. In China, serotypes A–F have all been detected in the soil (Yamakawa et al., 1988; Gao et al., 1990; Fu and Wang, 2008). In Japan, the presence of botulinum toxin serotypes B, C, and E has been documented (Yamakawa et al., 1988; Yamakawa and Nakamura, 1992; Umeda et al., 2013). In general, environmental botulism outbreaks have been connected to serotypes C, mosaic C/D, and E.

Less documentation is available regarding botulism outbreaks in natural environments within subtropical and tropical climate zones. On the African continent, *C. botulinum* has been detected in the soils of Zambia and Kenya, with identification of serotypes A–D (Nightingale and Ayim, 1980; Yamakawa et al., 1990; Karasawa et al., 2000). In Australia, the serotypes A, B, and D have been detected, either identified from cases of botulism or in soil (Eales and Turner, 1952; Murrell and Stewart, 1983; Koepke et al., 2008). In the tropical region of Indonesian waters, botulinum toxin serotypes A, B, C, D, and F were detected, but not serotype E (Suhadi et al., 1981). In the tropical Indian subcontinent, C and D are the predominant serotypes found in fish and aquatic environments (Lalitha and Gopakumar, 2000), and serotype E has not been detected (Lalitha and Surendran, 2002).

In the field of food safety research, laboratory studies have investigated spore resistance and factors favoring and limiting *C. botulinum* germination and growth—for example, the tolerated ranges for temperature, pH, and salinity (Chea et al., 2000; Hinderink et al., 2009; Derman et al., 2011; Stringer et al., 2011). However, the mechanisms triggering a botulism outbreak in the environment remain poorly understood. Several large-scale factors, such as lower water levels and/or higher summer surface water temperatures, have been correlated with larger outbreaks (Rocke et al., 1999; Perez-Fuentetaja et al., 2006, 2011; Lafrancois et al., 2011). Higher environmental botulism prevalences have also been reported when the sediment has a high organic matter content, the water has a pH of between 7.5 and 9.0, there is an overall negative redox potential, and the water temperature is above 20°C (Rocke and Samuel, 1999).

Pollution supports mass production of algae, followed by decay when packed ashore. In Lake Saint-Pierre, St. Lawrence River in Canada, years with low water levels coincided with eutrophic conditions and higher prevalence of filamentous green algae (Chlorophyceae), especially *Cladophora* (Cattaneo et al., 2013). Floating algae can create spots of strict anoxic conditions (Quortrup and Holt, 1941). In larger clearwater oligotrophic lakes in temperate climates, wind can cause circulation of surface water to a depth of 6–12 m or more. In the summer, this can expose the sediment surfaces within this depth range to temperatures of 10–20°C at an acceptable pH range for bacterial growth. Pollution of the nearshore waters can lead to developments as described for the great American lakes, in which massive shore accumulations of *Cladophora* served as biotic incubators for *C. botulinum* (Chun et al., 2013). Taken together, this observation indicate co-occurence between low water levels, growth of filamentous algae, and an increased risk of botulism.

A large number of samples from coastal waters and lakes have been investigated for the presence of *C. botulinum* strains or their spores. Many of these early studies were of importance for detection but provide superficial characterization of the localities sampled (Johannsen, 1963; Smith et al., 1978). Holomictic lakes in temperate regions may have two annual periods of circulation: immediately after ice-break and during the cooling period in autumn. A meso- to eutrophic lake may exhibit stagnation of the bottom water at a temperature near 4°C, with complete oxygen deficit during the late summer and late winter seasons. During seasonal circulations, this oxygen-deficient water is mixed in with the rest of the lake. In temperate regions where ice is uncommon, the lakes may be mixing and fully aerated through the coldest season. Brown-water forest/bog lakes and meromictic lakes (with permanent anoxic water at the bottom) may accumulate sinking organic particulates, crustacean exuvia, dead fish, etc. Although decomposition may be delayed by pH and/or low temperature, such lakes should be of interest as reservoirs of anaerobic bacteria. It is possible that some degree of vertical transport, upwards from suboxic or anoxic levels, may be mediated by resistant zooplankton with diurnal migrations, e.g., larvae of *Chaoborus* and some *Daphnia*. Overall, the yearly cycles of lakes may be relevant with regards to conservation and distribution of spores and substrates (Wetzel, 2001).

Extensive reed beds are found in shallow lakes in temperate climates, like Lake Balaton in Hungary and Lake Neusiedler See in Austria. Wildlife botulism has rarely been recorded from within the reeds. However, bird botulism has been reported in ponds on the shores of Neusiedler See (Zechmeister et al., 2005). In Spain, inland wetlands are more often troubled by bird botulism (Vidal et al., 2013) than coastal wetlands with a tidal regime (Contreras de Vera et al., 1991). This is probably due to both the water movement and the salt concentration. *C. botulinum* serotype C was less prevalent in seasonally flooded marshes than in permanently flooded marshes (Sandler et al., 1993). Furthermore, higher salinity has a negative effect on *C. botulinum* growth (Segner et al., 1971; Webb et al., 2007), decreasing the risk of botulism outbreaks.

## BIOTA AS RESERVOIRS AND VECTORS

*Clostridium botulinum* spores released into the environment are robust, potentially persisting in soils and sediments for decades (Long and Tauscher, 2006). The bacterium has been found in the intestinal tract of healthy fish, birds, and mammals. *C. botulinum* serotype E does not multiply in the fish gut (Bott et al., 1968), and fish fed 500,000 spores per day (in pellets) did not acquire botulism (Eklund et al., 1984). Thus, the initial proliferation of bacterial germination and vegetative growth must occur somewhere in the environment. Once established, a botulism outbreak is self-perpetuating. During an avian botulism outbreak, the disease spreads through necrophagous flies depositing eggs on dead and toxic animal carcasses. The resulting maggots feed on the carcasses and concentrate the botulinum toxin. When other animals ingest the toxic maggots, they become the next victims (the carcass–maggot cycle). During outbreaks in fish, decomposing invertebrates and decaying fish sink to the lake bottom and are consumed by scavenging fish in an amplifying cycle. A study of channel catfish showed that their lethal dose of botulinum toxin E was less than the median lethal dose for mice (Chatla et al., 2012). Toxin levels may persist and remain lethal over the winter in larvae (Hubalék and Halouzka, 1991). A wide variety of organisms—such as algae, plants, and invertebrates—have been shown to contain botulinum toxin or *C. botulinum* DNA (**Table 1**). These organisms represent a biotic reservoir for *C. botulinum*, and may themselves become toxic upon anaerobic decomposition (Quortrup and Holt, 1941; Heckman, 1986).

**Table 1:** *Clostridium botulinum* and possible vector organisms.

Environment	Vectors	Taxonomy	Sero-type	Area	Reference
Freshwater	Plants	*Ceratophyllum*	C	Norway (Oslo)	Skulberg and Holt (1987)
Freshwater	Plants	*Phragmites Schoenoplectus*	n.d.	Germany (Elbe estuary)	Heckman (1986)
Freshwater	Invertebrates	*Gammarus* (Crustacea), Oligochaeta (Annelida), Chironomidae (Insectae), Ephemeroptera (Insectae), Dreissenidae (Mollusca)	E	USA (Great Lakes)	Perez-Fuentetaja et al. (2006, 2011)
Freshwater	Algae, invertebrates	*Cladophora* (Chlorophyceae)	E	USA (Great Lakes)	Byappanahalli and Whitman (2009), Chun et al. (2013)
Wetlands	Invertebrates	Calliophoridae (Insectae), Ptychopteridae (Insectae), Hirudineae (Annelida), Isopoda (Crustacea)	C	Czech Republic (Moravia)	Hubalék and Halouzka (1991)
Wetlands	Invertebrates	Chironomidae (Insectae), Corixidae (Insectae), Sarcophagidae (Insectae), Calliophoridae (Insectae)	C/D	Spain (central Spain)	Vidal et al. (2013)
Wetlands	Invertebrates	Calliophoridae (Insectae), Coleoptera (Insectae)	C	USA (Utah)	Duncan and Jensen (1976)

For most of the insects listed, it is their submerged instars (e.g., mayflies) or larvae on carcasses (e.g., flies) that are vectors, with the important exception of the Coleoptera (beetles) and possibly the Corixidae (water boatmen).

It is clear that these organisms are involved in botulism outbreaks as part of the food web, and that birds and fish consume toxic decaying organic matter or toxic invertebrates, but little is known about the primary substrate in botulism outbreaks. Animals that die for other reasons but that contain spores in their digestive tract can serve as a substrate for bacterial germination. In the Great Lakes, invasive dreissenid mussels (*Dreissena polymorpha* and *Dreissena rostriformis bugensis*) and round gobies (*Neogobius melanostomus*; a benthic fish) have been suggested to contribute to the increased number of outbreaks by increasing the amount of decaying biomass (Getchell and Bowen, 2006). However, numerous other benthic organisms could potentially be responsible for transmitting *C. botulinum* to vertebrate prey organisms (Perez-Fuentetaja et al., 2011). During a disease outbreak in the Salton sea, PCR was used to test fish for serotype C-producing *C. botulinum*, but no difference in numbers of positives was detected among the groups of healthy, sick, and dead fish (Nol et al., 2004).

**FIGURE 1:**
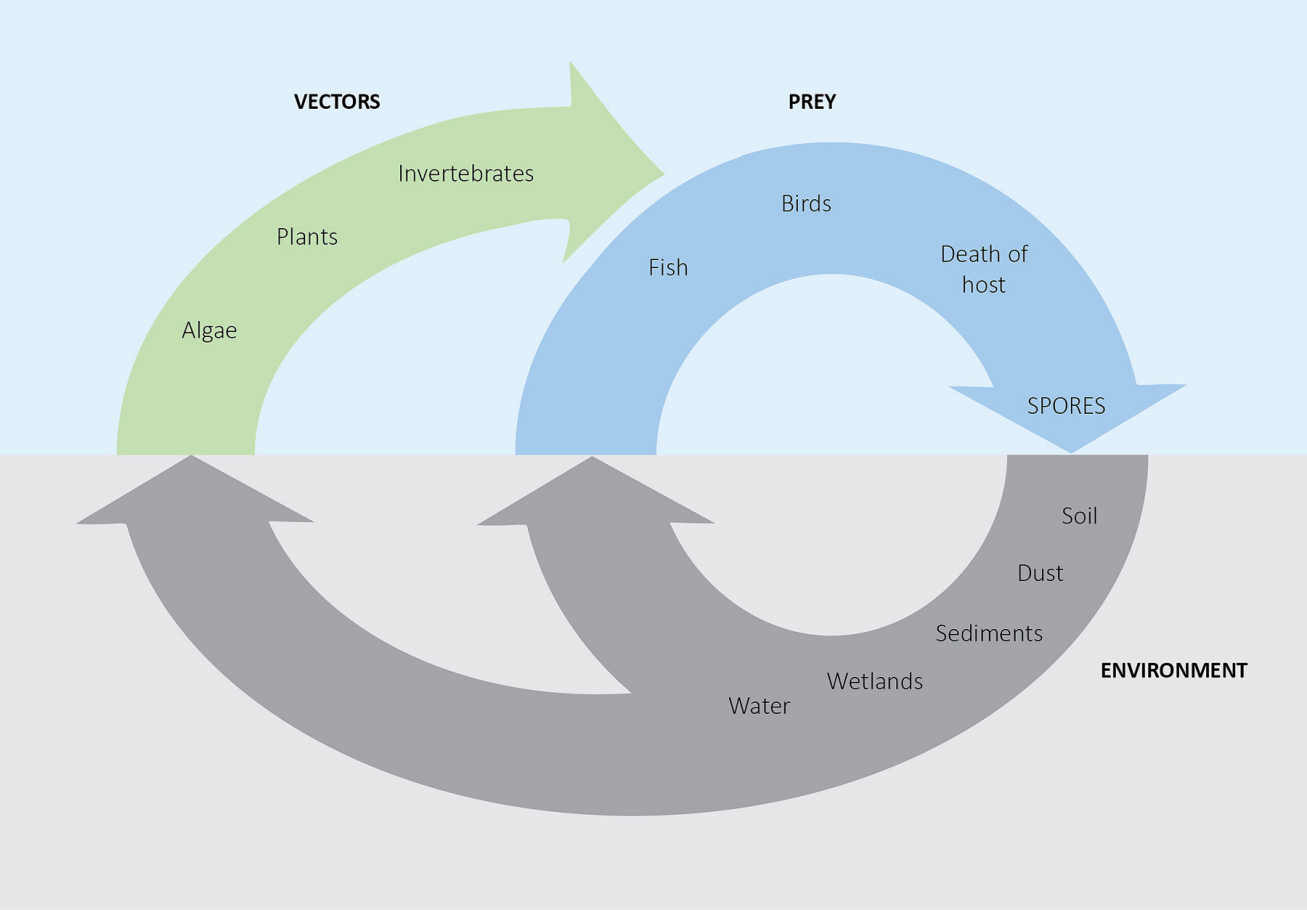
**Schematic representation of the role of *C. botulinum* in a freshwater ecosystem**. Resistant spores are released from dead hosts into the abiotic environment, and are spread by air and waterways. The spores may directly infect prey hosts or can be taken up by toxin-unaffected organisms, forming a biotic reservoir.

The filamentous green macroalgae *Cladophora glomerata* is reportedly associated with *C. botulinum* type E in Lake Michigan and Lake Ontario (Byappanahalli and Whitman, 2009; Chun et al., 2013). *C. glomerata* is globally widespread and can produce dense populations, especially under eutrophic conditions. Their high surface area is covered with organic compounds, which may form an ecological niche to diverse microbiota (Zulkifly et al., 2012). In floating algal mats, *C. botulinum* type E was found in high amounts of up to 15,000 cells (most probable number) per gram of dried algae (Chun et al., 2013). Heat treatment of *Cladophora* mat samples indicated the presence of *C. botulinum* vegetative cells (Chun et al., 2013). Another study analyzed senescent *Cladophora* samples from Lake Erie, and did not detect *C. botulinum* type E (Perez-Fuentetaja et al., 2011). An extensive survey revealed rich epiphytic microbiota on *Cladophora* thalli, but did not identify pathogenic bacteria, such as *C. botulinum*, associated with the alga and its epibionts (Zulkifly et al., 2012). The algae tested in this case were sampled from the attached macroalgae and not from floating decaying mats. In a laboratory experiment, sterilized plants and algae of different species, including *Cladophora*, were found to support anaerobe growth and toxin production of inoculated *C. botulinum* (Quortrup and Holt, 1941). The role of plants and algae as primary substrate for *C. botulinum* in wetland ecosystems must be further elucidated. **Figure 1** presents a schematic view of *C. botulinum* in a freshwater environment. It has been speculated that botulism outbreaks may be triggered by animals dying of other reasons than *C. botulinum* infection. Spores will germinate in the dead body, and after toxin production the carcass in the next turn is food for maggots and other invertebrates and an outbreak cycle starts. It remains to investigate/show if algal- and plant-associated toxin can start a botulism outbreak.

## CONDITIONS PREVENTING BOTULISM OUTBREAKS

Under natural conditions, there are a number of factors that can prevent *C. botulinum* growth. One limiting factor is the strong competition or even inhibiting effect by other bacteria (Smith, 1975; Girardin et al., 2002). Studies of marshland sediments have demonstrated inhibition of *C. botulinum* type C by other bacteria, including *Bacillus licheniformis*, *Bacillus mycoides/cereus*, *Streptococcus* spp., and *Clostridium* spp. (Smith, 1975; Sandler et al., 1998). Additionally, degradation of preformed botulinum toxin by aerobic bacteria has been experimentally demonstrated (Quortrup and Holt, 1941). In some environments, salt is a growth-inhibiting factor. Growth can also be reduced by lower temperature and pH, and acidification by fermentation (Quortrup and Holt, 1941). Fermentative processes of plant material in water by facultative anaerobic lactic acid bacteria (e.g., *Leuconostoc*) may initially create CO_2_, acetic acid and alcohol. However, as succession proceeds, the homofermentative species (*Lactobacillus* s. str.) take over and produce lactic acid, tolerating a lower pH (Buchanan and Gibbons, 1974; Giraffa et al., 2010).

One control measure that has been proposed to prevent outbreaks is to remove oxygen-deficient environments by raking the floating algae (Quortrup and Holt, 1941). Attempts have also been made to reduce the magnitude of botulism outbreaks by collecting carcasses, which appears to enhance survival compared to in areas with a higher carcass density (Evelsizer et al., 2010). It has been suggested that a functional ecosystems can better resist disease outbreak than dysfunctional ecosystems (Riley et al., 2008). An interesting research focus will be to further elucidate the mechanisms by which *Clostridia* are excluded, prevented, or outcompeted in many complex bacterial communities, in spite of favorable physical conditions, such as pH, salinity, and anoxia.

## *C. botulinum* AND CLIMATE CHANGE

An important question to discuss is whether climate change has or will contribute to increasing outbreaks of botulism. A study of the Salton Sea from 1907 to 1999 showed that avian diseases caused by various agents increased over the course of the 1990s (Friend, 2002). A study of Lake Michigan from 1963 to 2008 found a cyclic occurrence of outbreaks, with no increased frequency of outbreaks during the study period (Lafrancois et al., 2011). Since 1998, there have been yearly botulism outbreaks in Lake Erie, which have been spreading to other deeper Great Lakes (Perez-Fuentetaja et al., 2011). During dry periods, lower lake levels and high summer temperatures increase the growth of the filamentous green macroalgae *Cladophora* (Zulkifly et al., 2012), along with the risk of botulism outbreaks (Lafrancois et al., 2011). In the wetlands of central Spain, drought induced by overexploitation of groundwater resources represents an increased risk factor for local botulism outbreaks (Vidal et al., 2013). As climate forecasts predict warmer and wetter weather, in addition to more weather extremes, one may expect more outbreaks due to the warmer climate, especially if combined with prolonged dry periods and polluted water supporting blooms of benthic algae.

## Conflict of Interest Statement

The authors declare that the research was conducted in the absence of any commercial or financial relationships that could be construed as a potential conflict of interest.
